# Physician perspectives of the community paramedicine at clinic (CP@clinic) and my care plan app (myCP app) for older adults

**DOI:** 10.1186/s12875-024-02436-y

**Published:** 2024-05-25

**Authors:** Pauneez Sadri, Amelia Keenan, Ricardo Angeles, Francine Marzanek, Melissa Pirrie, Gina Agarwal

**Affiliations:** 1 Department of Family Medicine, McMaster University, 100 Main St W, Hamilton, ON L8P 1H6 Canada; 2 Department of Health Research Methods, Evidence, and Impact, McMaster University, 1280 Main St W, Hamilton, ON L8S 4L8 Canada

**Keywords:** Community paramedicine, Older adults, eHealth, Family physicians, Chronic diseases

## Abstract

**Background:**

Community Paramedicine (CP) is an emerging model of care addressing health problems through non-emergency services. Little evidence exists examining the integration of an app for improved patient, CP, and family physician (FP) communication. This study investigated FP perspectives on the impact of the Community Paramedicine at Clinic (CP@clinic) program on providing patient care and the feasibility and value of a novel “My Care Plan App” (myCP app).

**Methods:**

This retrospective mixed-methods study included an online survey and phone interviews to elucidate FPs ' perspectives on the CP@clinic program and the myCP app, respectively, between January 2021 and May 2021. FPs with patients in the CP@clinic program were recruited to participate. Survey responses were summarized using descriptive statistics, and audio recordings from the interviews thematically analyzed.

**Results:**

Thirty-eight FPs completed the survey and 10 FPs completed the phone interviews. 60.5% and 52.6% of FPs reported that the CP@clinic program improved their ability to further screen and diagnose patients for hypertension, respectively (in addition to their regular screening practices). The themes that emerged in the phone interviews were grouped into three topics: app benefits, drawbacks, and integration within practice. Overall, FPs described the myCP app as user-friendly and useful to improve interprofessional communication with CPs.

**Conclusions:**

CP@clinic helped family physicians to screen and monitor chronic disease. The myCP app can impact health service delivery by closing the gap between primary, community, and emergency care through an eHealth information-sharing platform.

**Supplementary Information:**

The online version contains supplementary material available at 10.1186/s12875-024-02436-y.

## Introduction

Community paramedicine (CP) is a new and developing community-focused health care model [1]. CP centres on advancing traditional paramedic roles beyond conventional emergency medical response [2]. The Community Paramedicine at Clinic Program (CP@clinic) is a chronic disease prevention, management, and health promotion CP program provided across Ontario and is expanding nationally and internationally [3]. The CP@clinic program trains paramedics to conduct individualized health assessments, provide health education, and refer patients to local community resources and back to primary care [3]. Paramedics apply evidence-based assessments using validated tools to screen patients for a variety of health-related risk factors [3]. Paramedics then share health evaluations with the patient’s family physicians (FPs) to enhance continuity of care [3]. CP@clinic patients are often vulnerable older adults (≥ 55 years) who are more likely to be frail due to their limited mobility and multiple chronic diseases [3, 4]. Recently, the CP@clinic program has been adapted to be delivered through in-home visits using the same CP@clinic assessments, called the ‘Community Paramedicine at Home’ (CP@home) program [3]. Patients in the CP@clinic and CP@home programs can be adults of any age and are often frequent users of Emergency Medical Services (defined as calling 9-1-1 at least four times per year) or at high risk of becoming frequent users [3].

To support CP@home, a patient-held eHealth intervention called the “My Care Plan App” (myCP app) has been developed and pilot tested with the goal of increasing communication, continuity of care, and program satisfaction between patients, physicians, and community paramedics. During the patient’s first CP@home program visit, patients typically undergo health behaviour, risk factor, and quality of life assessments, with two subsequent visits to monitor the patient’s progress and challenges. Following the launch of the myCP app, community paramedics would provide patients with a tablet that includes the pre-installed and configured app after performing the initial assessments. During the first visit, the paramedic will train patients to use the app. FPs can send patients and community paramedics actions or recommendations related to patients’ personalized risk assessments using the app. Patients and paramedics have the ability to record their actions, which assists patients in self-managing their health-related activities. This empowers patients to make independent and educated decisions while providing them with a sense of autonomy. This technology-assisted platform also benefits paramedics by providing a mechanism to seamlessly communicate with FPs, which can help close the gap between primary care, community care, and emergency care [5, 6].

Understanding how this app can promote patient continuity of care, develop seamless healthcare delivery, and improve disease screening, diagnosis, and management through communication and information sharing, is crucial before integrating or standardizing this eHealth intervention in any CP program. Thus, the purpose of this study was twofold; first, to determine the impact of the current CP@clinic program on patient screening, diagnosis, medication management, and health discussions around chronic diseases with FPs for CP@clinic patients; second, to evaluate FPs’ perceptions of the feasibility and value of the myCP app to support the CP@home program.

## Methods

### Design and setting

**Fig. 1:**
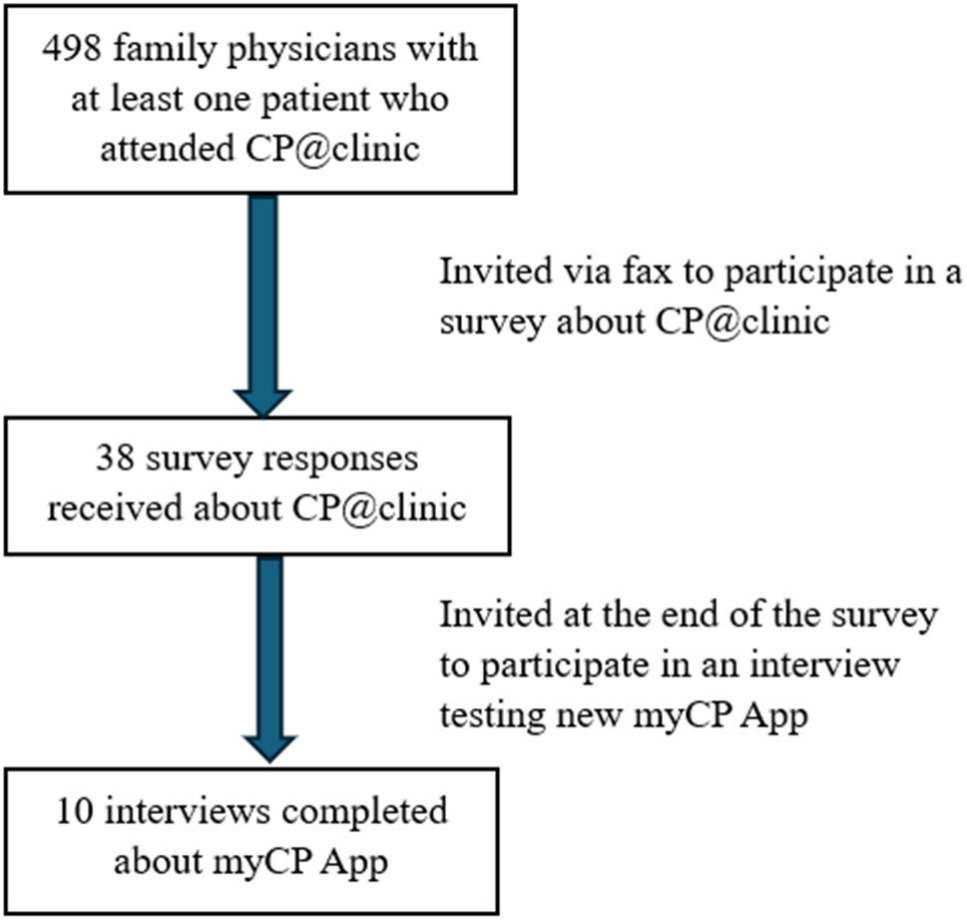
Study Flow Diagram

We performed a retrospective mixed-methods study consisting of two parts: (i) a survey to analyze FPs’ perceptions of the impact of the CP@clinic program on their patient management, and (ii) one-on-one phone interviews with FPs to gather feedback on the CP@home’s novel myCP app prototype. The survey was developed for this study by the research team (see supplementary file 1). The survey platform was the ‘Research Electronic Data Capture; (REDCap) application, a web interface used for managing online surveys [7]. Survey links were distributed between August 2020 and January 2021 to FPs. The survey also invited FPs who were interested in completing a Key Informant Interview (KII) over the phone, to share their perceptions of the newly developed myCP app. The interview guide (see supplementary file 2) was created by the research team and the phone interviews were conducted by research staff from January 2021 to May 2021 and lasted approximately 20 mins. At the time of the interview, a link was emailed to all interviewees to scroll through the app’s interface and test the app’s basic features. The interviewer also asked the FPs to complete tasks on the app (e.g. send patient messages, click on the patient’s next appointment) to familiarize themselves and practice using the interface before asking questions about the app. An honorarium was provided to FPs who completed the interviews. All interview recordings were transcribed using a transcription service. Please see Fig. 1 for a Study Flow Diagram.

### Sample and recruitment

Invitations to complete the surveys and interviews were only sent to FPs with at least one patient currently or previously enrolled in the CP@clinic program. A list of FPs who had been sent reports from the CP@clinic database was compiled by the CP@clinic database administrator, and then a fax was sent to those FPs inviting them to complete the survey via a unique REDCap survey link. FPs were faxed with invitations up to four times if they did not respond to previous faxes. Consent to participate was provided electronically, and upon completion of the survey, FPs received an honorarium in the form of a gift card by email. This study was approved by the Hamilton Integrated Research Ethics Board #​​14645.

### Data Collection

Demographics were collected independently for FP participants who completed the survey on RedCap and for FP participants who completed the phone interviews. The survey consisted of ten questions on how CP@clinic had influenced their practice and took approximately five minutes to complete. The survey used multiple-choice style questions and participants could select multiple answers for each question. Specifically, the survey asked FPs if the CP@clinic program had increased or improved their test or screening practices for diabetes, hypertension, and falls. The survey also asked about CP@clinic’s ability to improve their diagnosability, medication initiation or adjustments for diabetes and hypertension, and if CP@clinic facilitated or increased patient discussion for other chronic diseases or case coordination. Finally, a yes-no question asking FPs if they would recommend the CP@clinic program to other physicians was included. The phone KIIs were semi-structured with questions that allowed FPs to discuss what they liked about the interface, their concerns, changes they would make to the app, and how they would integrate the app into their daily practice. Phone interviews were led by research staff and recordings were later transcribed.

### Data analysis

Quantitative data from the surveys were analyzed using descriptive statistics with reported frequency measures. Qualitative data from the interview transcripts were thematically analyzed in September 2021 through iterative coding following an initial thematic analysis by a research team member [8]. Three other independent team members analyzed the transcripts and validated the themes that were initially coded, adding to the themes and sub-themes iteratively. A series of meetings were held to discuss the thematic concepts to demonstrate that necessary rigour was applied to the coding. A thematic codebook was created to group the various themes, subthemes, and direct quotes. Themes and subthemes were verified against the transcripts repeatedly until all research team members were confident that they appropriately captured the content.

## Results

A total of 498 FPs were identified from the CP@clinic database and were faxed survey invitations; of these, 38 responded. Survey respondents varied in age, gender, years of practice, and community of practice; survey respondent characteristics are summarized in Table 1. The majority of participants were over the age of 45 (*n* = 21, 55.3%), and more than half of the participants had been practicing as an FP for over 11 years (*n* = 22, 57.9%).

**Table 1:** Summary of Physician Characteristics who completed surveys

Characteristic		Physician Survey Participants *N* = 38 *n* (%)
Gender	Male	18 (47.4)
	Female	18 (47.4)
	No Response	2 (5.3)
Age (Years)	25–34	5 (13.2)
	35–44	10 (26.3)
	45–54	14 (36.8)
	55–64	7 (18.4)
	No Response	2 (5.3)
Years of Practice	Less than 5	4 (10.5)
	5–10	9 (23.7)
	11–15	3 (7.9)
	16–20	8 (21.1)
	More than 20	11 (28.9)
	No Response	3 (7.9)
Community of Practice (categorized by Ontario Health Regions)	Central Region	7 (26.9)
	West Region	8 (30.8)
	North East Region	2 (7.7)
	No Response	9 (23.7)

A total of 10 FPs who completed the survey also agreed to participate in the phone interviews to provide their perspectives on the myCP app. A summary of the interview participants’ demographics is shown in Table 2.

**Table 2:** Summary of Key Informant Interview Physician Characteristics

Characteristics		Number of Participants *N* = 10
Gender	Male	5
	Female	3
	No Response	2
Age	25–34 years	0
	35–44 years	2
	45–54 years	3
	55–64 years	3
	No Response	2
Years of Practice	Less than 5 years	1
	5–10 years	0
	11–15 years	1
	16–20 years	1
	More than 20 years	5
	No Response	2

### Survey results

The survey results are presented in Table 3. The most common aspect of care that FPs reported benefiting from the CP@clinic program was related to hypertension; 60.5% reported improvements in their testing/screening for hypertension, 52.6% in their ability to diagnose hypertension, and 57.9% in medication initiation/adjustment for hypertension. Although reported less frequently, some FPs did find the program beneficial for providing care related to diabetes, primarily for medication initiation/adjustment (15.8%) and testing/screening (15.8%), which is a meaningful impact. Nearly one-third also reported that the program facilitated discussions with their patients about other chronic diseases and facilitated care coordination. When asked if they would recommend the CP@clinic program to other physicians, of those who responded to this question (*n* = 35), 94.3% of FPs responded yes (*n* = 33).

**Table 3:** Descriptive analysis of physician responses to the CP@clinic for Physicians Survey

Responses to the CP@clinic for Physicians Survey	
**Benefits of the CP@clinic Program in Patient Care**	
**Aspect of care improved**	**Frequency** *N* = 38 **n (%)**
Screening for Diabetes (*n* = 38)	6 (15.8)
Screening for Falls (*n* = 38)	4 (10.5)
Screening for Hypertension (*n* = 38)	23 (60.5)
Diagnosing Diabetes (*n* = 38)	2 (5.3)
Diagnosing Hypertension (*n* = 38)	20 (52.6)
Initiating or Adjusting Medication for Diabetes (*n* = 38)	6 (15.8)
Initiating or Adjusting Medication for Hypertension (*n* = 38)	22 (57.9)
Facilitating/Increasing Discussions about Chronic Diseases (*n* = 38)	11 (28.9)
Facilitating Case Coordination (*n* = 38)	10 (26.3)
Facilitating care for Other Health Conditions/Issues (*n* = 38)	5 (13.2)
**Recommendation of CP@clinic Program**	
Would recommend the CP@clinic program to other physicians (*n* = 35)	33 (94.3)

### Key informant interview results

Ten major themes were identified in the KIIs. These themes were combined into three topics with similar or related areas which include: (1) app benefits, (2) potential app challenges, and (3) app considerations for integration within practice. Within the ‘app benefits’ are features FPs liked about the app. ‘Potential app challenges’ include themes related to anticipated difficulties. ‘App considerations for integration within practice’ include themes that target changes to the app to align with FPs’ roles and practice. Each of the ten themes and their furthered divided subthemes is shown in Table 4.

**Table 4:** Illustrative quotes from the key informant interviews

**Topic 1: App Benefits**	
***Theme 1. User-friendly App Organization and Layout***	
(A) Technology Accessibility	“… Sometimes the older patients tend to be a little bit more challenged by technology… So that’s why we need to keep it simple and, you know, a tablet-based thing would be easy to train as opposed to a smart phone or an app on a smart phone.” (KII 7)
(B) Concise layout	“It’s very clear in terms of their risk, so I like how that has been put on the screen, I guess. It’s very clear what the issues are. Easy to read, it’s short and to the point.” (KII 8)
(C) Usability	“ I liked that… what the patient screened positive for was in red, so that was great, that really stood out. And it seems it was organized quite well. You know, they gave the tasks for the patient is very clear to me what they’ve been asked to do. It tells me, did the patient complete the task, yes, when the next appointment is with the paramedic, because that’s great because then I can, you know, if there’s something that’s changed with the patient that the paramedic might not know about but is close and I can send that information and say, okay, this is now what’s changed.” (KII 2)
***Theme 2. Asynchronous Communication***	
(A) Time Efficiency	“I think it’s very efficient timewise. You know, we’re very pressed for time as family physicians, so I don’t have to schedule an appointment or make a phone call or … I can type a message to the patient or the paramedic and hit “Submit” and it doesn’t take more than a minute.” (KII 1)
(B) Goal-Oriented Communication	I mean we always have them in the office and we just tell them to lose weight and that, but I mean it would be nice to give them specific guidelines, aims, targets for weight, weight reduction and all that. I think they would be able to expedite that with the patient probably better than we could. It would be more efficient. (KII 3)
(C) Virtual Care	“I like the concept because I think the way it’s going right now, especially with COVID, a lot of stuff is being done over the phone now and, you know, not direct patient contact in the office. So I like that the patient is at their home or out of the office. It’s more informal, which I’ve always preferred anyways. I always liked house calls because there was no time limit, there was no, you know, appointment, you just went in there and did what you had to do. “ (KII 3)
(D) Monitoring	“I think it would be great for an older patient who is either on their own or with their spouse who is also older, just for keeping an eye on medical problems. So for high blood pressure, it’s great if people are instructed on how to measure their blood pressure effectively, appropriately, and monitoring it for themselves and for myself is useful. Well, I think that has been a helpful aspect of having the paramedics go to see the patients. (KII 5)
(E) Mobile Restrictions	“Well, I can think of, especially in the pandemic patients, elderly patients who don’t want to leave their building, this way they can get a health assessment without having to leave their building…” (KII 1)_
***Theme 3. Increases Dialogue and Assessment***	
(A) Holistic Assessment	“… I think flagging issues at home that may be related to their surroundings; so mobility or … low activity. So something that might be evident in the home that I can’t see when I see a patient in the clinic… the same thing pertaining to their diet, to kind of take a look at, you know, what’s in the household, that type of information can be really helpful because sometimes you don’t get a sense of that in a clinic.” (KII 5)
(B) Identifying Risk Factors	“… I like that it identifies some of these kind of lifestyle things which, like I said, in a busy practice you may not have time to ask about. And it would probably bring to life some stuff that I don’t know about my patients because someone has taken the time to [ask]…that’s a game changer. So this is probably definitely some practice changing kind of information.” (KII 9)
(C) Follow-Up appointments	And, I think having the next visit date is helpful too, that you can get a sense of, okay, maybe I’ll, you know, hear something back in that time frame. (KII 5)
(D) Social Housing Patients	“… people in subsidized housing have a tendency to have less interactions with their family physicians just because of time, or cost, and so they fall through the cracks.” (KII 4)
(E) Increased Paramedic communication	“… And there’s discourse between the frontline worker and the physician.” (KII 3)
**Topic 2: Potential App Challenges**	
***Theme 1. Physician Liability***	
(A) Allocation of Responsibility	“Well, if this is all I got, there would need to be some follow-up with the paramedic. So I would need to say, look, you gave me this information, I need more, I need more clarity as to each one of these that you’ve asked about because now we’re medically responsible for this data gathering and so if this [app] says… the patient has possible problems with depression but if that problem involves suicidal ideation and that’s been brought to my attention through this…[app] I become medically responsible for it; so I’d probably have to reach out to the paramedic and say, what do you mean by this, and what has been done.” (KII 9)
(B) Severity Level of Patient Information	“I think if it was easy to access and, you know, as long as you could relay information that was, you know, low risk or low probability for harm if it wasn’t acted upon, it would be fine. But I wouldn’t trust it to make a medication dose change…” (KII 5)
(C) Information Sharing	So, I’m not sure how…patient information is going to be protected …but that needs to be in place …[so] someone else can access [the app] if I’m unavailable. (KII 2)
***Theme 2. Patient Hesitancy***	
(A) Technological Literacy	“ I think the only thing I would wonder for some patients, whether they would be—some of them are elderly, and whether they would be motivated to use the app or be a little bit intimidated by it. But I suppose if it was really straightforward like this, I could see it being useful.” (KII 8)
(B) Patient Confidentiality and Privacy	“Maybe patient confidentiality, I guess. For some patients, they’d want to know who has access to this information” (KII 1)
**Topic 3: App Considerations for Integration within Practice**	
***Theme 1. App Accessibility for Physicians***	
A) Integration within EMR	“So if it was received like through my regular EMR, where we get faxes and, you know, messages and lab results and imaging results sent to us, if it was integrated somehow and if it was easy to access, then I think it would be useful.” (KII 5)
(B) Notification Integration	“… If I can be notified somehow of this link but that it could be, you know, somehow come to the office instead of just to me in case there’s something that. can’t wait, let’s say a week, that I should be dealing with…” (KII 2)
***Theme 2. Changes to App Layout***	
(A) Prioritization of Patient Data	“…so yeah, definitely there needs to be a triaging… I think some of the stuff at the bottom certainly should be grouped together and kind of kept at a different tier almost as the ones that are a bit more concerning, like the alcohol intake. And so there should be a bit of a triaging for this report so that the high-level things are highlighted for you and then the like red flags, yellow flags, you know, green flags….so that we have an idea of where should I spend most of my attention.” (KII 9)
(B) Group Similar Patient Data Together	“…The layout of the page on my screen there’s a lot of empty space. If they could just move everything closer together so that it’s on a single page.” (KII 4)
(C) Separating Paramedic and Physician Responsibilities	“… this is a bit lengthy to read through. And so if there was two sections, one of: here’s all the things that we found but we’ve taken next steps for and versus here’s all the things we found that require your input…” (KII 9)
***Theme 3. Additional App Features***	
(A) Date of Last Assessment	“I would love to know what the date of the assessment was so that you can get an idea of how old this information is.” (KII 4)
(B) Medication Compliance	“I think probably a list of medications somewhere might be helpful of the patient, what the patients actually have and take, are two different things. The amount of naturopathic and over-the-counter medications aren’t captured in their visits to the office but there it can be easily listed…” (KII 7)
***Theme 4. Patient Assessments and Guidelines***	
(A) Tailored/Individualized Guidelines	“… even within the hypertension guidelines, there will be different tolerability levels for each age group, right…so it shouldn’t necessarily be flagged the same way as. if it was a younger patient.” (KII 9)
(B) Screening Tool Validity	“The other thing I would wonder about when I’m looking at this whole thing is, are these screening tests validated…” (KII 9)
***Theme 5. App Usability for Physicians***	
A) Easier Communication	“I think more than anything [I would use the app to] contact the paramedic… if the paramedics are following, [the patient] multiple times a week, I think it would just be more clear in terms of what the goals are…I think that information I have gotten from community paramedics has been pretty useful; so I think if it was in this interface, I would use it.” (KII 8)
B) Patient Information Dissemination	“Yeah. This is helpful for the risk factors. I mean, to know about their alcohol intake, you know, diet, salt, salt intake, that’s not stuff that we always have time to ask about in our visits with them, you know. Usually it’s catered towards specific chief complaints. So for someone to have gone through and figured okay, they’re doing less than 30 min of physical activity, I would hope that some of these kind of lifestyle changes the paramedic would be able to offer kind of some next steps…” (KII 9)

## App benefits

The three themes that emerged under app benefits were: (1) user-friendly app organization and layout, (2) asynchronous communication, and (3) increased dialogue and assessment.

### User-friendly app organization and layout

Most FPs described the organization and layout of the app to be clear, concise, and simple to read. FPs noted that providing older patients with a tablet with the app already installed was more convenient than asking them to download a novel app on their phones. The app would be easy for them to use since it was organized in clear sections for reading health guidelines and monitoring health targets. The app’s organization was also thought to be helpful for FPs to quickly identify patient risk factors, specifically that the app highlighted in red each health problem for which the patient screened positive. The app displayed the patient’s next appointment and patient tasks very clearly, which helped FPs navigate patient updates at a quick glance. Most FPs were pleased with the aesthetic features like the fonts, colours, organization, and lay language used to present information to patients. It was important to all FPs that the app’s interface allowed them to quickly identify key patient information while also anticipating that it would be easy to read for older patients who may be hesitant to use this technology initially.

### Asynchronous communication

Several FPs described the myCP app as being an effective tool to communicate with their patients asynchronously, particularly because it could help them communicate with patients and community paramedics despite their busy schedules. FPs believed that using the app was a more time-efficient way to communicate with paramedics or patients instead of waiting for paramedics to fax the patient’s screening report or waiting to discuss a patient’s health problem at their next appointment. FPs also described the myCP app as being a useful tool for paramedics or physicians to disseminate specific health targets or guidelines (e.g. for weight, blood glucose, blood pressure) to patients that they could constantly refer to on their own. They described how once the myCP app sends the FP the patient’s initial assessments, they should be able to quickly follow up with the paramedic by providing them with tailored patient health targets. FPs liked that in addition to providing them with targets, it could help instruct patients on how to measure or assess risk factors on their own (e.g. measure blood pressure). This is helpful for older adults living alone and who lack assistance or the knowledge to monitor changes to their health. Further, using the app to communicate with patients and paramedics to screen or monitor risk factors was aligned with the FP’s virtual care model and remote services integrated within their practice throughout the COVID-19 pandemic. FPs noted that their patients living in social housing frequently missed their appointments due to mobility and transportation issues, preventing FPs from scheduling follow-up appointments. Thus, the app is ideal for FPs with these patients to communicate with the paramedic and remotely monitor patient health.

### Increased patient dialogue and assessment

A majority of the FPs identified that the most beneficial outcome of the myCP app would be its ability to help them identify their patients’ risk factors in a timely manner. Given that paramedics would be visiting patients at their homes and discussing the patient’s overall health (as opposed to a primary complaint that needs to be treated at a clinic), they would be able to better assess the patient’s home environment, diet, mobility, and overall quality of living.

Since patient information would then be instantaneously communicated through the myCP app, FPs could conduct a more holistic patient assessment using information that FPs would not be able to monitor, have time to ask or have access to when at their clinics. This would allow FPs to make a more comprehensive assessment of the patient’s health and provide them with treatments that would be tailored to their lifestyle and environment. This would also help tailor the frequency of appointments relative to the needs and urgency of a patient’s specific conditions, allowing for more efficient use of the FP and patient’s time. FPs also noted that certain patients might be more comfortable speaking to paramedics in their homes, particularly those that had difficulties travelling or accessing a clinic’s in-person services. FPs reported that patients living in social housing often had trouble completing health assessments at the clinic and they were less likely to report their health problems that could alternatively be highlighted in discussions with the paramedic. FPs reported that having their next patient’s appointment available to them on the app would help resolve the issue of having to ask administrative staff about their patients’ appointments, or not knowing when the patient would be seen next. The app would also allow for increased communication between the paramedic and the patient. Throughout the regular CP@clinic and CP@home programs, FPs noted that community paramedics could not send personalized messages or communicate to FPs besides faxing them screening report results. As both community paramedics and FPs play integral roles in a patient’s circle of care, FPs liked that the app’s organization would provide a platform for all members of the patient’s circle of care to easily and frequently communicate with each other.

## Potential app challenges

Two themes were identified related to potential app challenges: patient hesitancy and physician liability.

### Patient hesitancy

FPs noted that patients may be reluctant to use a new piece of technology - even if the organization and layout of the interface were simple and easily accessible. Even though FPs were told that patients are not required to input any values or information on the app themselves, they were still concerned about introducing new software and teaching patients how to use it. FPs anticipate that patients may also have concerns over patient confidentiality and would want to know who else has access to their app profile and how their health information would be protected. FPs also expressed concern when told that the app contains a separate section, not visible to the patient, for the FP to communicate with the community paramedic on how to manage the patient’s health. One FP commented that any information shared with the community paramedic concerning the patient’s health and care plan should also be shared and available for the patient to see.

### Physician liability

Several FPs mentioned that one of their main concerns with the myCP app was the responsibility and liability attached to their role within the app. For example, when receiving patient updates from the community paramedic on the app, FPs want to know if they are medically responsible for screening for emergency-related issues, calling the hospital if any data proves to be concerning (e.g. suicidal ideation), or if they can receive further clarification from the paramedic. Another concern was whether or not other physicians or specialists could log on to the app if the FP was not available to read new messages or patient updates. Overall, however, FPs noted that this app would be useful if the information was not time-sensitive or if the information being relayed to the FPs was low risk given their limited time availability and occupied schedules. In addition, several FPs questioned the type of information the community paramedic would send them. FPs felt that it was not clear whether the information that community paramedics would send them was for them to review or for them to act upon and that this should be made more clear on the app. Finally, many FPs expressed that while the app would be a helpful tool, FPs are already responsible for documenting patient information in electronic medical records (EMRs) and thus are already overwhelmed with the number of software programs and emails that require monitoring. If the app was an additional software that required a unique login and password, many FPs would be hesitant to integrate the interface into their daily practice.

## App considerations for integration within practice

The ‘integration within practice’ topic resulted in four themes: accessibility, patient guidelines, additional app features, and feedback from community paramedics.

### Accessibility

One of the main challenges FPs noted about integrating the myCP app into their everyday practice was the increase in duties to manage and monitor patient information in a new application. Thus, FPs suggested that to decrease this burden of responsibility, the myCP app could be combined or consolidated with the patient’s EMR. Other FPs suggested emailing them links or finding a different way to ensure that the app was not a new software that would add more work to their daily workflow. Lastly, FPs discussed that it would be helpful to incorporate app notifications by email or fax to better monitor or stay accountable for any patient changes or updates.

### Additional app features

Although most FPs liked the layout and aesthetic of the app, some FPs suggested grouping patient information by severity. This would ensure that essential patient details are displayed first or presented differently from the rest of the data. Although the myCP app presents patients’ health problems in red, other FPs noted that using a more comprehensive colour-coding system (e.g. highlight information in green once complete or for less critical information) would be helpful. This will help FPs triage information by severity or urgency and allow them to follow up on more significant patient health risks without reading the entire patient’s profile. FP also requested changing the app’s layout to include accordion-style menus that reveal additional information after it has been selected instead of showing all the patient’s data on one page (which can appear cluttered or overwhelming). In addition, although the app displays the patient’s next appointment, FPs expressed that it would be helpful if the app could show the patient’s last screening or health assessment with the paramedic to view the patient’s health timeline or history. Almost all FPs wanted the app to include a section on medication compliance to track which medication patients were actually taking because most of their patients (particularly those living in social housing) lack transparency on their medication intake. Community paramedics would be better equipped to observe medication compliance in a patient’s home and report any changes through the app.

### Patient assessments and guidelines

Regarding the available guidelines for each patient (e.g. hypertension, blood pressure guidelines), FPs want the app to consider the variability amongst different kinds of patients and their health needs. Each patient should have access to specific guidelines on their health depending on their personalized health risks and chronic conditions. One FP felt it was important to know how the screening tools have been validated. Suggestions were made to include references or detailed explanations of the screening tools that CP@clinic is using to demonstrate to physicians how these tools are evidence-based.

### Communication with paramedics

Using the app to specifically communicate with paramedics to send or receive updates on their patients will allow for more time-efficient and targeted patient follow-ups. This interface also helps FPs by quickly displaying their patient’s goals and targets based on the other health information on the patient’s app profile. Additionally, it was expressed that the app would be most applicable to screen patients for risk factors that the paramedic could more quickly and conveniently evaluate when visiting patients at their homes. This would allow FPs to make a more comprehensive patient assessment and use health data typically not assessed at the patients’ scheduled appointments.

## DISCUSSION

The current study found that, from the FP perspective, having patients enrolled in the CP@clinic program improved the FP’s ability to provide healthcare, especially for chronic diseases. It also found that adding the myCP app to the CP@clinic program would be well-received by FPs based on their review of the current app prototype.

### Impact of CP@clinic on physician care

In summary, the survey highlighted that in addition to FPs’ usual practice of care, CP@clinic improved FPs’ ability to screen, diagnose, and initiate/adjust medications for hypertension and diabetes, and increase health discussions with patients in the program. Based on their experience, 94.3% of FPs would recommend the CP@clinic program to other physicians. This finding is significant since physicians report that providing care to their patients can be challenging without having up-to-date data from appropriate risk assessments [9]. Therefore, receiving this risk assessment data from the community paramedics, who use validated tools, can help the physician provide high quality care.

The benefits of CP@clinic reported by FPs in the current study aligns with an RCT which found that blood pressure significantly improved in CP@clinic attendees that had high blood pressure on their first visit, and that diabetes risk in attendees significantly decreased over time [4]. One feature of the CP@clinic program is that attendee risk assessment information is sent to the family doctor through regular reporting (or same-day communication if more urgent concerns are identified) [4]. The findings of the current study suggest that FPs were able to act on this information and it had an impact on their ability to provide care for patients with hypertension or at risk of diabetes.

### Feasibility of the My Care Plan app (myCP app)

#### Benefits of the myCP app

Overall, the FPs in this study were pleased with the myCP app prototype, were willing to use it, and supported its role in a community paramedicine program. The FPs in our study specifically described the app as aesthetically pleasing and user-friendly with features that allowed for efficient dissemination and communication between paramedics, physicians, and patients. One study found that community paramedics often find the most challenging aspect to systematically integrating CP into Ontario’s health care system is successfully communicating with physicians and initiating or engaging in these relationships [2, 10]. Community paramedics have suggested developing comprehensive communication strategies within clinical care models and ensuring they can individually contact their patients’ FPs [10]. It is clear that enhanced communication between all members of the patient’s circle of care, including the patient, can lead to improved patient care. The myCP app can help achieve this goal by allowing FPs and community paramedics to update each other on the patient’s health assessment and care plan.

#### Potential challenges of the myCP app

Our study showed that FPs were concerned about their legal liability to check or report on the patient’s information on the app and on the security and privacy of their patient’s information. Physicians in another eHealth study mentioned similar concerns, however, since the data from third-party apps is not part of the patient’s EMR, these physicians viewed this data as supplemental information that can help increase patient health behaviours, which can reduce physician liability surrounding this data [11]. Similar to our study, physicians, health organizations, and other eHealth app developers are hesitant to create or recommend apps to older patients due to their lack of technology literacy [11]. A recent study suggested that apps that help users recover quickly from mistakes, use feedback messages, include user options to return to previously searched information, and use clear video instructions to register and use the app are more successful with older adults than those that don’t include these features [12].

### Integration within practice

While the feedback on the myCP app was very positive, FPs did suggest several functions or actions that could improve the integration of this software within their practice. Changes to the app interface to ensure customization or prioritization of patient data by severity and physician responsibility and credible tailored patient guidelines were mentioned. Similarly, other physicians have regarded ‘usability’ to be an essential component of their experience, as well as frequent progress feedback, app customizability, usability, and credibility [11, 13]. FPs in our study also wanted to ensure the myCP app could be integrated with other apps or EMRs that they use daily. Physicians in other studies are also highly keen on app integration within patient medical records [11]. In a recent study, 68% of physicians said that efficient app integration of collected data into EMRs would increase the likelihood that they would implement information from health apps in their practice [14].

Another study found that if issues such as data privacy, quality standardization, and legal implications of physician roles in using these apps were properly addressed then physicians would be willing to use health apps more intensely and commonly with their patients [15]. Overall, FPs in our study are most keen to use the myCP app to communicate with and increase dialogue between themselves, the community paramedics, and their patients.

### Implications of the mpCP app and future directions

The integration of technology (i.e. the use of mobile phones, and tablets) within health care programs and services has drastically increased within the past 10 years [16].Literature shows that integrating mobile phones into elderly patients’ healthcare management led to increased medication compliance, treatment adherence, and improved daily life management through monitoring, which are clear benefits for this population of elderly living independently or in isolation in urban areas [4, 17]. Many CP@clinic and CP@home patients are seniors living in social housing buildings and have lower health literacy, therefore adding the user-friendly myCP app to these programs could be beneficial for patient care and health outcomes [18].

An incidental finding from the KIIs was that a majority of FPs were unaware or even surprised when informed about the benefits of CP. This is consistent with existing literature describing that FPs have had limited interactions with community paramedics and are unfamiliar with their scope [4, 9, 14]. As a result, the extent to which community paramedics are integrated into the patient’s circle of care is often limited [14]. To increase the impact of the CP@clinic and CP@home programs, FPs need to gain a deeper understanding and awareness of the roles and responsibilities of community paramedics, which can ultimately facilitate improved engagement, communication, and patient care [10, 17].

The myCP app has the potential to impact health service delivery by closing the gap between primary, community, and emergency care through an eHealth information-sharing platform. If integrated into the CP@home program, this technology will support integrated care for vulnerable populations whose chronic conditions are often not effectively managed [19]. Coordinating patient care will help reduce the costs and burden on emergency services and improve health management by increasing communication and information sharing between FPs and community paramedics [20]. In addition, the myCP app can help FPs create partnerships or integrate care in the community and help assist public health policymakers to advocate for the increased use of eHealth applications in CP programs. Future studies on this topic should aim to explore the perspectives of patients and community paramedics on integrating the myCP app within their CP programs. This would provide a more comprehensive understanding of how the app can be integrated into the patient’s primary care plan. It can also examine which app features patients and paramedics are more likely to benefit from. Future research can also be directed at comparing CP@home assessments before and after using the app to see if it improves patient health over time. Specifically, a study can be conducted to assess the improvement of patient quality of life measured using the Euroqol 5 Dimensions (EQ-5D) [21] for a pre-post intervention assessment.

### Study strengths and limitations

There are several strengths to this study. The KIIs provided in-depth views of FPs practising in Ontario with patients currently in a CP program; this is an uncommon scenario and it is difficult to get the attention of FPs for such a study. In addition, FPs were asked their opinions exactly at the time they viewed the app and not afterwards, therefore there was no delay in response leading to memory difficulties.

There were also limitations to this study. First, the CP@clinic survey for FPs had a small sample size. Recruitment for the survey was challenging as several fax notifications were sent to FPs with patients in the CP@clinic program; however, very few responded. Second, the respondents who agreed to take part in the KIIs were all FPs practicing in Southern Ontario, which may not capture the perspectives of all FPs or primary care professionals. Third, the data collected from the surveys and the KIIs was specific to the CP@clinic program and myCP app, which may limit generalizability; however, similar CP programs or other health care programs seeking to integrate an app may be able to apply these findings. Finally, the semi-structured nature of the interviews implies that specific questions were asked in follow-up to the comments made by some FPs and not others. This could have provided an imbalance in the type and amount of information that was extracted throughout the KIIs.

## Conclusions

In conclusion, FPs perceived CP@clinic to be beneficial to their practice, especially when providing care for patients with chronic diseases. In addition, the KIIs underscored several positive impacts that the myCP app could have on identifying patient risk factors and increasing dialogue between community paramedics and FPs. The app was found to be user-friendly and recommendations were made to facilitate seamless integration of this app within the professional roles of FP and CPs.

### Electronic supplementary material

Below is the link to the electronic supplementary material.

Supplementary Material 1

Supplementary Material 2

## Data Availability

The datasets generated during the current study are not publicly available to ensure confidentiality of participants but are available from the corresponding author on reasonable request.

## Abbreviations

CP: Community Paramedicine
FP: Family Physician
CP@clinic: Community Paramedicine at Clinic Program
myCP app: My Care Plan App
REDCap: Research Electronic Data Capture
KII: Key Informant Interview
EMRs: Electronic Medical Records
EQ-5D: Euroqol 5 Dimensions Questionnaire
