# Supplementary material for: Physician perspectives of the community paramedicine at clinic (CP@clinic) and my care plan app (myCP app) for older adults
Source: BMC Prim Care. 2024 May 25;25:187. doi: 10.1186/s12875-024-02436-y (PMC11127385; doi:10.1186/s12875-024-02436-y)
Supplement: Supplementary file 1 — Supplementary Material 1 [file 12875_2024_2436_MOESM1_ESM.docx]

**Survey Questions for Physicians of CP@clinic Participants Impact Survey**

1. Community where your practice serves:
2. Gender:
   1. Male
   2. Female
   3. Other
3. Age
   1. 18 to 24 years
   2. 25 - 34 years
   3. 35 - 44 years
   4. 45 - 54 years
   5. 55 - 64 years
   6. 65 - 74 years
   7. 75 years and over
4. Years of practice
   1. Less than 5 years
   2. 5 to 10 years
   3. 11 to 15 years
   4. 15+ years
5. Has CP@clinic increased/improved your: (Please check all that apply)
   1. Test/screening practices for
   2. Hypertension
   3. Diabetes
   4. Falls
6. Has CP@clinic increased/improved your: (Please check all that apply)
   1. Diagnosis for:
      1. Hypertension
      2. Diabetes
   2. Medication adjustments/initiation for:
      1. Hypertension
      2. Diabetes
7. Has CP@clinic:
   1. Facilitated or increased patient discussion for other chronic diseases
   2. Facilitated case coordination
   3. Other:
8. Would you recommend the CP@clinic program to other physicians?
   1. Yes
   2. No

**Separate Form**

1. Are you able to participate in a 20 minute telephone interview with research staff to help us improve CP@clinic as we develop a patient-held and physician interactive interface?
   1. Yes
   2. No
2. Thank you for completing the survey.
