# Supplementary material for: Physician perspectives of the community paramedicine at clinic (CP@clinic) and my care plan app (myCP app) for older adults
Source: BMC Prim Care. 2024 May 25;25:187. doi: 10.1186/s12875-024-02436-y (PMC11127385; doi:10.1186/s12875-024-02436-y)
Supplement: Supplementary file 2 — Supplementary Material 2 [file 12875_2024_2436_MOESM2_ESM.docx]

In the interview today, we will discuss a physician interface we are developing to exist alongside an extension of the CP@clinic program, a mobile app called MyCarePlan app, or myCPapp.

Each participant of CP@Clinic will receive a tablet with the myCPapp on it. The myCPapp will enable participants to track their goals and health resources to address these goals. The myCPapp will be connected to the interface to inform you about your patient's risk assessment and individual tailored health education by the paramedic. You will be able to provide input or comment on it. Your patient will be able to see what you write. The interface also allows you to give advice to the community paramedic on how to manage this patient that will not be visible to the patient.

**Do you have any questions about how this works before we begin with the questions?**

Ok, would you be able to pull up the link I sent in the email yesterday? It can be on your phone or a computer.

As you browse through the interface, we will ask you some questions:

1. First I am going to give you a few tasks to carry out so we can test the usability of the interface. Take your time to find the answers.

- What are 3 of the participant’s chronic disease risk factors?
- When is the participant’s next appointment?
- What are the 3 tasks given to the participant by the paramedic?
- Tell the participant that you recommend reading Canada’s Food Guide to reduce their salt and fatty food intake.
- Tell the paramedic your patient set a goal to walk more during their last doctor’s appointment.

1. How would you see yourself using this interface to manage your patient?
   1. Review patient’s risk factors?
   2. Contact paramedic?
   3. Contact patient?
2. What do you like about this interface and why?
3. What other information would you want to know about your patient?
4. What would you change about the interface, if at all? And why?
5. What concerns, if any, do you have about this interface?
6. Thinking of your patients, can you discuss an example of how the interface may be used?
7. How do you see this interface being integrated into your routine?
   1. What would be easiest for you? Email? Fax? EMR?
   2. How would notifications about patient updates best work for you?

Ok thank you, I just have a few demographic questions to finish off the interview.

What is your gender?

Male

Female

Gender Diverse

Other

What age group do you fall between?

Less than 25 years

25 - 34 years

35 - 44 years

45 - 54 years

55 - 64 years

65 - 74 years

75 years and over

How many years have you been practicing?

Less than 5 years

5 to 10 years

11 to 15 years

16 to 20 years

More than 20 years

Ok thank you very much, that concludes the interview.
